# The Beat

**Published:** 2008-06

**Authors:** Erin E. Dooley

## The Green Gap

A product may be greener than the competition, but that doesn’t mean the product actually improves the environment. Consumers don’t always grasp the distinction, however, as illustrated by the *2008 Green Gap Survey*, released in April 2008 by the Boston College Center for Corporate Citizenship and Cone LLC. The survey showed that while 39% of respondents prefer to use products marketed as “green” or “environmentally friendly,” nearly 48% misinterpret these terms as synonymous with “environmentally beneficial.” This so-called green gap can lead consumers to believe products are “friendlier” than they actually are. Nearly half the respondents reported trusting companies to accurately portray the environmental impact of their products, a fact that Center for Corporate Citizenship director Bradley Googins says “may suggest the lack of control they feel around complex environmental issues.” The FTC is currently reviewing guidance to help marketers avoid making inaccurate or misleading environmental claims.

**Figure:**
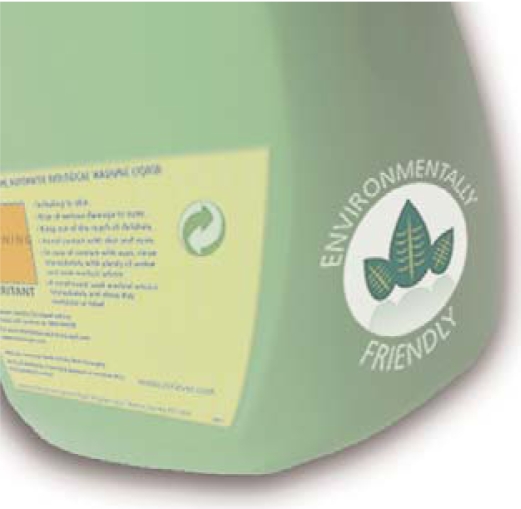


## Metal Transformation

The U.K.’s Peak District, once a center of metal mining, is now an important recreation area, with an estimated 16 million people living within a 1-hour drive. Scientists reported at the April 2008 annual meeting of the Society for General Microbiology that the district is home not only to numerous rare animal and flower species but also to metal-eating bacteria, whose excretions may convert heavy metals in the soil into more soluble toxic forms that can leach easily into groundwater, reservoirs, and waterways. Furthermore, scientists believe changes in soil bacterial composition could help free carbon from the district’s peat bogs, which store an estimated 24 metric tons of the material per square kilometer per year.

**Figure:**
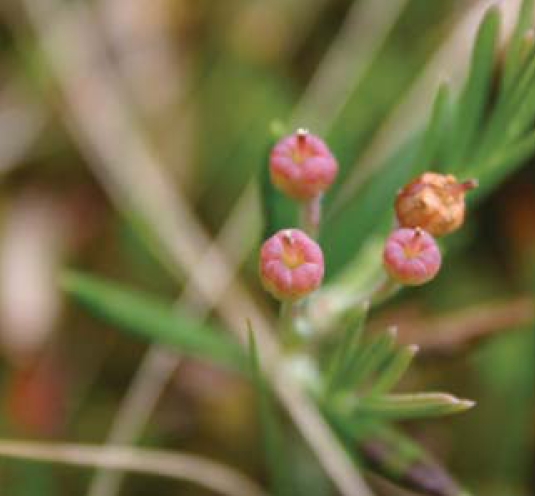
Bog rosemary, a Peak District native

## Imaging FlowCytobot Nips Algal Blooms in the Bud

The Imaging FlowCytobot, an automated underwater cell analyzer developed at Woods Hole Oceanographic Institution (WHOI), is set to make beaches safer as the water warms up. The Imaging FlowCytobot quantifies and photographs microscopic plants in the water. The images are electronically relayed to shore-based laboratories, where software classifies the plankton. Researchers recently used the device to detect an unusual species of harmful algae in the Gulf of Mexico in time to prevent a shellfish poisoning outbreak in humans. This development comes just in time—marine surveys and new computer models also developed at WHOI forecast unusually large blooms of the toxic algae *Alexandrium fundyense* for New England in 2008.

## The Wonder Pollutant?

Single-walled carbon nanotubes (SWCNTs) are 10,000 times thinner than a human hair, stronger than steel, and more durable than diamonds—properties that have earned them the title of “wonder materials.” But health and environmental advocates want more research on these materials before they spread any further in commerce. In the 7 May 2008 issue of *Nanotechnology*, scientists report that, unlike previously assumed, various commercially produced SWCNTs display vastly different compositions, which will make tracing their impact on human health and the environment more difficult. The authors conclude that failure to establish an understanding of the chemistry of these materials could result in unintended environmental consequences, eventually leading to product bans and expensive cleanup efforts like those associated with industrial materials such as asbestos.

**Figure:**
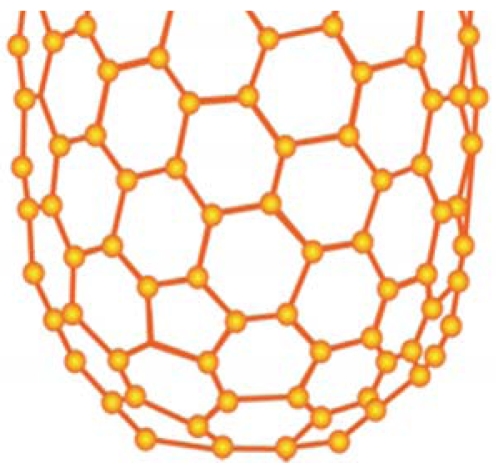
A mesh of carbon nanotubes

## House Tackles Invasive Species

Hundreds of nonindigenous aquatic species are unintentionally introduced into U.S. waters through discharged ballast waters from oceangoing vessels, with a new invasive species being identified about every 28 weeks. These hitchhikers cause economic and ecologic degradation to affected near-shore regions. On 24 April 2008, the U.S. House of Representatives passed HR 2830. Under the bill, oceangoing vessels have until 2013 to install ballast treatment equipment that can exceed by 100 times new international standards for the removal of invasive species. The bill also would establish a program to evaluate alternative ballast water treatment methods and an initiative to study methods for monitoring and controlling aquatic species spread by routes other than ballast water. At press time a companion Senate bill (S 1578) had not been voted on.

**Figure:**
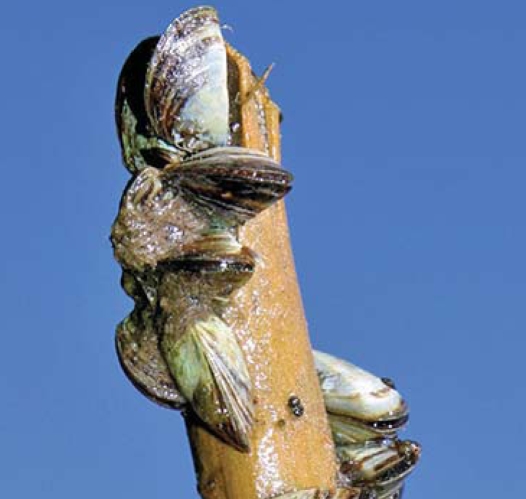
Invasive zebra mussels cluster on a stick pulled from Charleston Lake, Ontario

